# Are goals scored just before halftime worth more? An old soccer wisdom statistically tested

**DOI:** 10.1371/journal.pone.0240438

**Published:** 2020-10-20

**Authors:** Henrich R. Greve, Jo Nesbø, Nils Rudi, Marat Salikhov

**Affiliations:** 1 INSEAD, Singapore, Singapore; 2 Independent Researcher, Oslo, Norway; 3 Yale School of Management, New Haven, Connecticut, United States of America; Instituto Politecnico de Viana do Castelo, PORTUGAL

## Abstract

There is an old soccer wisdom that a goal scored just before halftime has greater value than other goals. Many dismiss this old wisdom as just another myth waiting to be busted. To test which is right we have analysed the final score difference through linear regression and outcome (win, draw, loss) through logistic regression. We use games from many leagues, control for the halftime score, comparing games in which a goal was scored after 1 minute remained of regulation time with games in which it was scored before the 44^th^ minute. Our main finding is that the home team scoring just before halftime influence these outcomes to its advantage, compared with scoring earlier with the same halftime score. We conclude that a goal scored just before halftime has greater value than other goals provided it is scored by the home team. In other words; the wisdom may be old, but it’s still wise.

## Introduction

The economic importance of soccer [1] and the social consequences of soccer results [2, 3] have led to significant research interest in how soccer games are played and won. Much of this has focused on confirming or dismissing beliefs held by so-called soccer experts (trainers, players, media, and fans). One old wisdom that has not been getting much attention among sports analysers until recently is whether a goal scored just before halftime is of greater importance for the end result than other first half goals. Commentators and coaches of some of the best soccer teams in the world have made statements that indicate that they do believe these goals being disproportionally important. For example, Jürgen Klopp; “We scored in the perfect moment … before … before halftime … it was absolutely perfect.” (8th December 2018) and the World Cup Final Commentator; “Zidane! Lightning has struck twice… What a time to score a second.”(12th July 1998). Others we interviewed do not believe this to be the case, including Ole Gunnar Solskjær, currently managing Manchester United and Egil Olsen, who managed Norway’s national team when it reached number two on the FIFA World Ranking.

Theoretically the effect of such goals matters because it shows the effect of cognitions and emotions on judgment and behaviour. Cognitively, a success event such as a goal leads to less alert processing and a temporarily decreased performance [4], which is less consequential if the goal occurs before a break. Emotionally, a goal increases confidence in the scoring team, but more importantly, for the team that concedes the goal it can lead to quick learning and recovery if the team is experienced in mutual adjustment [5, 6]. This quick learning can be interrupted by a break, impeding the recovery of the team that concedes the goal. Consistent with this, behavioural psychologist Jan Ole Hesselberg says goals just before halftime *feel* more important because they have more emotional impact, so they stay with us during the break. They are therefore stored in our memory in a different way, which in turn may lead to a confirmation bias [7, 8]. Managers, media commentators, and players may believe that goals just before halftime affect second half performance even in the face of little evidence. On the other hand, Hesselberg does not rule out the confirmation of our bias. Even beliefs founded on limited evidence can hold true.

As sports analysis is getting to be more commonly consumed and discussed among soccer experts and fans, it seems we are getting used to old wisdom becoming busted myths proven wrong by statistical analysis. For example, the myth that sports of concentration are won by the player that is least affected by performance variability is contradicted by evidence that golf players at any level show loss aversion, just as everyday people do [9]. The “hot hand” belief that players in many sports get better scoring rates after a string of scores has been debunked in some sports studies but supported in others [10–12].

Of course, the idea that a first half goal scored late becomes more important may be the result of any combination of confirmation bias, accurately recorded observations, experience, beliefs in psychological mechanisms and others. But it is not obvious that we would automatically and based on naïve ideas about the mental effects conclude that to score a late goal and then have a break is advantageous. In some ways, quite the opposite. Old wisdom in tennis—which like soccer is a sport of action and the opponent’s response—is that when you are scoring, you should keep up a high tempo between serves, thus avoiding any breaks in which the opponent has the time to rethink their strategy. This would imply that there is a bigger chance that the playing pattern remains the same, a pattern in which you seem to be the better player. Although tennis has a different scoring system (1–0 is not enough, you have to keep on scoring to win), if it’s true that changing the tactics is more advantageous in a sport of action and response, you would expect a goal just before halftime to be *less* worth.

Still, could scoring just before halftime be one of the cases where the numbers actually agree with the old wisdom? We will investigate this question by defining a goal just before halftime (a “late goal”) as a goal scored in or later than the 44^th^ minute of the first half and examining its effects on the final goal difference and game outcome—loss, tie, or win.

Whether this old wisdom is true or not has consequences beyond soccer. In our lives we are surrounded by organizations that create the products and services (private and public) that we depend on, and organizations use teams both in their daily work and for important projects such as creation of new and improved offerings and correction of errors. Boeing currently has teams for new product development and for improving the design of the 737 MAX aircraft that has had its second sudden crash. Teams often encounter setbacks and need to adjust to these through self-organized changes and/or through management or coaching by supervisors. A soccer team is composed of players who are experts with years of training, and they have a manager (coach) who will give halftime instructions and motivation. For them to be influenced by the timing of opposing team’s goals suggests that team adjustments to setbacks have a certain fragility. Either the team’s members or the manager is doing something differently in a way that harms the performance, and if it can happen in teams with this level of expertise, it surely happens elsewhere also. Alternatively, the team that scored the goal may benefit from a break shortly after, which would be similarly surprising given their level of expertise.

Three earlier studies have directly or indirectly looked at the empirical value of goals just before half time [1, 13, 14]. None of these found evidence that certain timings of previous goals influenced final game outcomes beyond the goal itself. Baert and Amez’s study [1], the most recently published and the one closest to our study, examined whether the final outcome of a soccer match was disproportionately influenced by whether a goal was scored in the final minutes before halftime. Surprisingly, the study found weak negative effects for home team goals just before halftime (i.e., scoring late goals was a disadvantage for the home team) and mostly insignificant effects for away team goals [1]. This finding appears to disprove the old wisdom. However, as the authors note, the data are limited to two specific competitions, the UEFA Champions League and Europa League, and the competing teams that meet in these competitions vary along many dimensions. This raises the question of whether the findings can be replicated based on analyses based on a large data set from a range of competitions. Indeed, a recent working paper analyses data from five European leagues, comparing goals scored at the end of the first half with goals scored at the beginning of the second half and finds that goals before are more beneficial for the scoring team [15]. This analysis compares two very different time intervals, thus potentially conflating end-of-period and start-of-period effects, and it is also based on a rather small dataset of 1,930 games. Although closer to our analysis, the highly specific time intervals compared in [15] is a significant limitation as compared with an analysis of all alternative goal scoring times.

The objective of this study is to re-examine these conclusions with datasets that are larger and have less unobserved heterogeneity. We analyze the effect of scoring just before halftime where even the most restricted dataset has 60 times more games than the data used in [1]. Our motivation is simple. First, we are not sure that the data used in [1] are enough to draw conclusions. Second, teams in national leagues are more familiar with each other and the stadiums they play in, and they face (national) playing styles and referees drawn from a national pool. This amounts to holding constant many factors that vary across games in the international UEFA matches. From a statistical viewpoint, that would likely reduce the error term in the model because there are fewer unmeasured influences and more observations. From a soccer viewpoint, it means that we are using data on games in which the approach to playing games is more routine and well-known to players and coaches. This could imply that the goal scored just before halftime comes as a greater shock to the conceding team and is a greater boost to the team that scores. There is still the possibility that the many leagues we use for our estimates differ from each other, so we take such differences into account for our modelling.

## Materials and methods

### Sample

Our study is based on data collected from the World Football data web site (www.worldfootball.net), which has goal timing and game outcomes for more than 300,000 games from European and American national leagues and the web site betexplorer.com, which has historic betting odds. We restrict our data to the top leagues in each nation and include only the games for which we have betting odds available, which leaves 72,426 games for analysis from year 1998 to year 2016 from 27 top-level national leagues listed in S1 Appendix. We denote this dataset WF-WFC.

In addition, we use *BA*, the dataset on UEFA Champions League and Europe League that has earlier been used to test the effect of goals just before halftime [1]. This dataset was used as supplied from the authors with no alterations. We also use *WF-BA*, a dataset drawn from Worldfootball that mimics the data-collection procedures of [1], but it has more observations and produces slightly different findings ([1] excluded 186 games without "a substantial competitive value", i.e., the result makes no difference for advancing, but we do not have the list of these games, so WF-BA also includes them). We also use *WF-L*, a dataset with all leagues in Worldfootball without requiring the betting odds, which gives the maximum number of observations at the cost of not controlling for team strength. This data has no matching or filtering. If we filter this dataset by limiting it to the top leagues but still do not restrict it to having betting odds we get *WF-LT*. Finally, our largest possible dataset includes all available games without any merging of betting odds or limiting the time period or league; this is *WF-MAX93*.

S1 Table shows the descriptive statistics for these datasets.

### Procedure

Both www.worldfootball.net and www.betexplorer.com are commercial web sites with reliable data, and we were able to use their data without additional processing. To obtain the dataset WF-WFC we matched the data from the two sites based on the date of the game and fuzzy matching of team names, with two team names considered equal if their normalized Levenshtein similarity was above 0.5. This threshold allows us to match most of the games while avoiding false matches. We also filtered the data. The full Worldfootball data have multiple leagues in some nations, but we limit our data to the top league in each nation to ensure that the data consist of the most elite clubs. Lower leagues may have greater variability in team quality and many (but not all) of them are also missing betting odds.

To our knowledge, this is the largest dataset generally available for the type of analysis needed to answer this question with sufficiently high-level play, and sufficiently evenly matched teams to give the issue of late-goal advantages practical importance. After all, finding poor decision making or other forms of unusual team performance is of less interest in teams that are inexperienced, or when one team is engaged in the exceptionally difficult task of trying to beat a much better team.

### Data analysis

We estimate models in which the dependent variable is either the goal difference in the end of the game (away goals—home goals) or the ordinal game outcome for the home team (win < tie < loss) as the dependent variable. In both models, a negative coefficient means an *advantage* to the home team. The goal difference models are linear regression estimated through ordinary least squares, and the game outcome models are estimated by ordinal logistic regression (also known as ordered logit), both using R software. The table reports p-values from two-sided tests of whether the coefficient estimate equals zero.

### Variables

In some models we control for the relative strength of the two teams by using the betting odds for the match. Betting odds can be used as a “wisdom of the crowds” assessment of team strengths as they are in part set dynamically to balance the “book” of bets, and thus they represent the prices in a market. Prediction markets have been found to have excellent properties in general [16, 17] and specifically for soccer games [18, 19]. Betting odds can be transformed into goal expectancy for each team through a Poisson model [20], and we use this procedure to generate the variables λ_h_ and λ_a_, which are goal expectancies for the home and away team, respectively.

## Results

**Fig 1:**
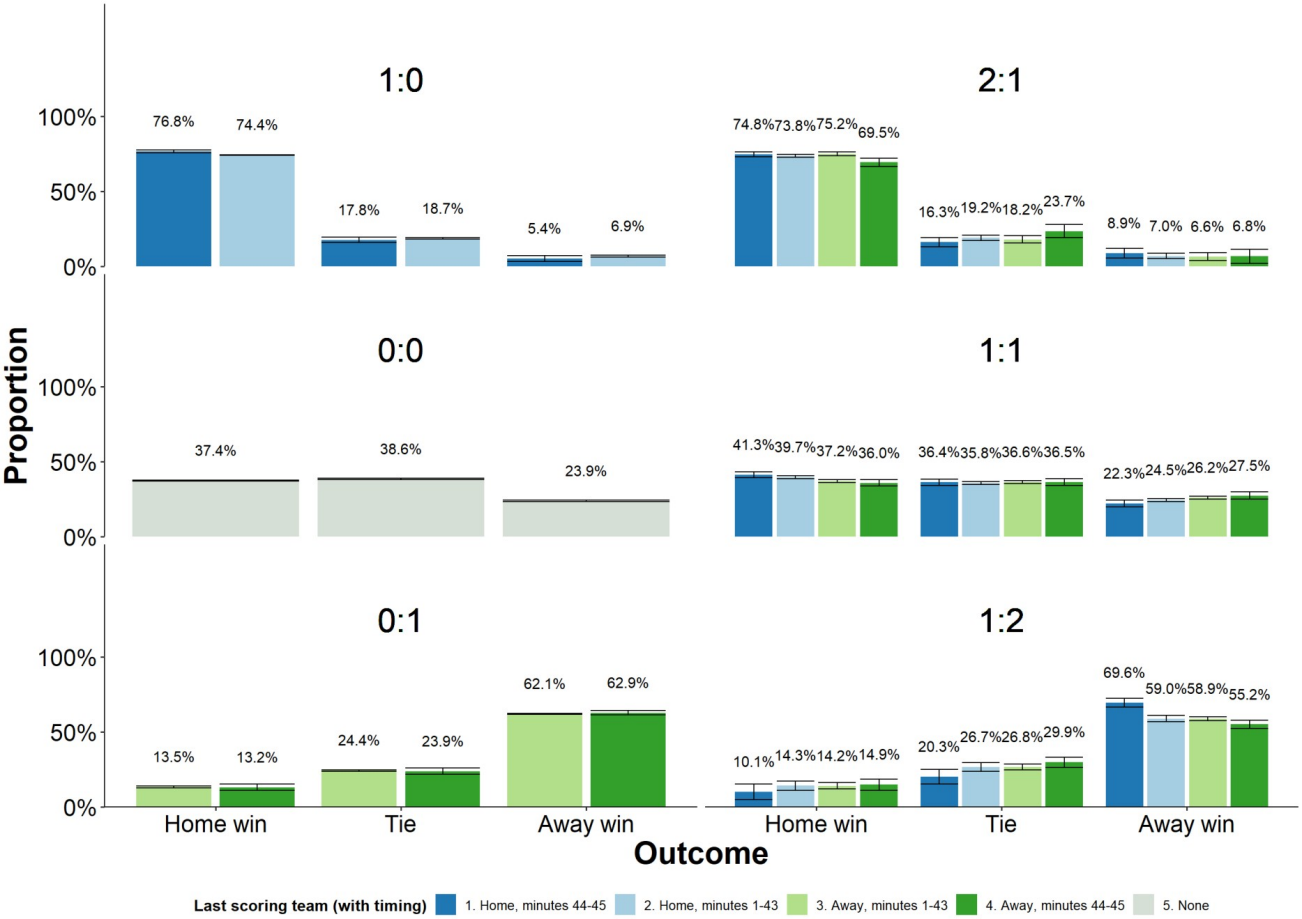
Proportions of home, tie, and away final results for different halftime scores.

Fig 1 demonstrates how outcomes for six of the most common halftime results depend on who scored the last goal and its timing. The rows represent home lead, tie, and away lead. In each graph, the columns show from left to right home goal but not last minute, away goal but not last minute, away goal last minute, and no goal. The 0:0 plot is a reference giving distribution of home, tie, and away outcomes if no team scored in the first half. For ease of interpretation, the dark color indicates games in which a goal was scored just before halftime. The graphs with two or fewer goals before halftime have the most observations, with more than 10,000 each, while 1:2 and 2:1 must be interpreted with caution because they are based on far fewer observations.

The halftime score of 1:1 is of particular interest to our investigation because it means that the teams are tied by halftime, so the only difference is in the order and timing of the goals. The findings strongly suggest that goals just before halftime are special. The likelihood of the home team winning slopes downward in the four cases: last-minute home team goal, home team goal last, away team goal last, and last-minute away team goal. The proportion of a tied game shows no significant differences among these conditions, and the proportion of an away team win slopes upward. The graph suggests that if the halftime score is 1:1, any last-minute goal will be more influential for the final result than earlier goals.

The scores 1:0 and 0:1 also suggest that a last-minute goal is more influential than an earlier goal, but the effect appears weaker in these games. This is reasonable because the halftime score often reflects a real difference in team strength, and the team that is behind after halftime has only 45 minutes to reverse the score. The 2:1 and 1:2 figures have fewer observations and opposing results, with 2:1 indicating that home-team last-minute goals benefit the home team, but 1:2 indicating the opposite. As mentioned, these should be interpreted with caution because they are based on fewer observations.

Fig 1 is suggestive but not conclusive. We need to precisely estimate the effect of late goals, and its standard deviation, to assess our confidence in the visual difference. Next, we turn to regression analysis of the same data, using OLS regression of the score difference at the end of the game (away-home) and ordered logit analysis of the outcomes in view of the home team win—tie—loss.

Table 1 shows the results of the analysis using the main dataset WF-WFC. The last-minute goal is especially influential in some conditions but not others. We see that in 1:1, it affects the final goal difference and game outcome at the 1 percent significance level only if the late goal is by the home team. The effect is increased score difference and likelihood of a favourable outcome for the home team, just as the old soccer wisdom claims. A last-minute goal in 1:0 also affects the second half goal difference and likelihood of a favourable outcome as the soccer wisdom claims. For 0:1 there is no significance, repeating the 1:1 finding that away-team late goals do not have significant effects. For the high-scoring halftime results, the last-minute goals have effects opposite of the soccer wisdom, but these findings appear unduly influenced by the small number of observations with variable half-time goal differences. The main results of the analysis are (1) the late-goal advantage is found for home team goals in games with halftime result 1:1 and 1:0, (2) the late-goal advantage is not found for away teams with halftime results 1:1 and 0:1, and (3) the goal difference at halftime is more influential than the timing of the goal for 1:0 and 0:1 halftime results.

**Table 1:** Full-time results predicted by halftime goal difference (WF-WFC).

	*Dependent variable*:					
	Away goals—Home goals				Outcome	
	(1)	(2)	(3)	(4)	(5)	(6)
Goal home	-1.023***	0.064***	-0.884***	-0.881***	-1.504***	-1.433***
	(0.015)	(0.016)	(0.014)	(0.014)	(0.023)	(0.023)
Goal away	1.058***	-0.029*	0.924***	0.927***	1.509***	1.444***
	(0.017)	(0.018)	(0.016)	(0.016)	(0.024)	(0.024)
Goal sequence HA	0.028	0.028	0.041	0.045*	0.052	0.069*
	(0.027)	(0.024)	(0.025)	(0.025)	(0.035)	(0.036)
Goal sequence AH	-0.017	-0.017	-0.002	-0.005	-0.046	-0.032
	(0.026)	(0.024)	(0.025)	(0.025)	(0.034)	(0.035)
Remaining^+^ X home last	-1.889***	0.030	-1.643***	-1.635***	-2.084***	-1.913***
	(0.019)	(0.023)	(0.018)	(0.018)	(0.034)	(0.035)
Remaining^+^ X away last	1.448***	-0.0001	1.264***	1.270***	1.634***	1.493***
	(0.022)	(0.023)	(0.021)	(0.021)	(0.033)	(0.034)
Difference of Goals		1.087***				
		(0.009)				
λ_h_ home goal expectancy			-0.783***	-0.589***		-1.074***
			(0.016)	(0.022)		(0.029)
λa away goal expectancy			0.747***	0.617***		1.005***
			(0.020)	(0.026)		(0.035)
Goal home X late goal	-0.093**	-0.093**	-0.071*	-0.068*	-0.140**	-0.121*
	(0.041)	(0.038)	(0.039)	(0.039)	(0.069)	(0.071)
Goal away X late goal	0.050	0.050	0.049	0.042	0.031	0.031
	(0.050)	(0.046)	(0.047)	(0.048)	(0.072)	(0.075)
Goal sequence AH X late goal	-0.117*	-0.117**	-0.114**	-0.098*	-0.085	-0.091
	(0.062)	(0.056)	(0.058)	(0.058)	(0.080)	(0.082)
Goal sequence HA X late goal	0.042	0.042	0.021	0.008	0.057	0.043
	(0.063)	(0.057)	(0.059)	(0.059)	(0.081)	(0.083)
Remaining^+^ X late home goal	0.127***	-0.006	0.112***	0.106***	0.272***	0.275***
	(0.035)	(0.032)	(0.033)	(0.033)	(0.064)	(0.066)
Remaining^+^ X late away goal	-0.253***	-0.052	-0.202***	-0.210***	-0.369***	-0.316***
	(0.044)	(0.040)	(0.041)	(0.042)	(0.064)	(0.065)
Team and competition FE	No	No	No	Yes	No	No
Observations	72,426	72,426	72,426	72,426	72,426	72,426
R^2^	0.333	0.453	0.414	0.429		
Adjusted R^2^	0.333	0.453	0.414	0.415		

Notes:

^+^All scores except 0:0,1:0,0:1,1:1

*p<0.1;

**p<0.05;

***p<0.01

Next, we analyse a wide range of datasets to examine the robustness of these conclusions. S2 Table shows the findings. The BA dataset (UEFA Champions League and Europe League) contrasts to our findings. Models (2) and (4) are estimated on these data and show the sign of the regressor indicating a disadvantage to the home team when scoring in the last minute. The sign change and insignificance are the same as shown in [1]. The results of all other datasets are consistent with the home team benefitting from scoring a last-minute goal in the first half. The findings are significant and remarkably consistent across the different datasets. Only minor differences in effect size distinguish the models.

In summary, across a broad range of datasets and model specifications we find that a late goal in the first half is more advantageous than a goal scored at another time, provided it is scored by the home team rather than the away team.

The models using data from national leagues unambiguously demonstrate that the earlier finding of a late-goal disadvantage for the home team is specific to the BA data using only limited data from Champions League and Europe League. It cannot be reproduced drawing a larger number of games from the same leagues from the Worldfootball database (in fact, the sign of the effect becomes opposite). Furthermore, it is contradicted by the analyses using the extended data from the national leagues, either only top leagues or all leagues in the database, as these all show a significant late-goal advantage to the home team. The old soccer wisdom of the late halftime goal advantage is supported by the analysis, but significantly so for home teams only.

## Discussion and conclusion

This study is a follow-up and correction of three earlier studies that directly or indirectly looked at the empirical value of goals just before half time [1, 13, 14], one of which found evidence that certain timings of previous goals influenced final game outcomes beyond the goal itself [1]. In particular, Baert and Amez’s study [1], the most recently published and the one closest to our study, found weak negative effects for home team goals just before halftime, contrary to the common belief that the goals just before halftime are particularly beneficial. We conducted this analysis again using larger datasets and more familiar teams, as we relied on the top level national leagues, and found the opposite result. Home team goals just before the halftime gave a greater advantage than home team goals at other times, whereas for away team goals the timing had the opposite directional effect—not with statistical significance. These findings are conclusive.

So why is a goal just before halftime more important?

It is not within the scope of this paper to answer that question, but a promising start is to listen to the insiders of the game. It is interesting that all the coaches, players and fans we interviewed emphasized the psychological effect, the mental boost and positive energy that a late goal brought to the locker room during the break—even the interviewees who did not believe in the extra effect of goals just before halftime(!). Does positive energy—however that is defined—and a feeling of having been rewarded, produce better results than the opposite feeling? And if so, why is it better to have a break right after that reward instead of continuing playing? Maybe it isn’t. Maybe any goals give the “mental boost” that the experts we interviewed suggest. The nature of a boost is that it is temporary, so let’s imagine it lasts for the next ten minutes. In that case the boost after an early goal will have evaporated when it’s time for the break, while the same goal scored just before the break will mean that the scoring team—if it can contain all or some of this “boost” during the break—have a relative advantage when the second half starts. Conversely, the team conceding a late goal enters the locker room on a negative note. Does this this negative feeling settle during the halftime break, whereas it would dissipate if they could continue playing? Does it trigger unwise coaching decisions? Our findings suggest that it is problematic for the away team conceding a late goal.

Clearly, the following sequence of events underlies the findings. The goal is scored just before halftime, and the teams enter the locker room without much play following the goal and with a fresh memory of the goal. In the locker room, the team is assembled in a meeting rather than spread out on the pitch as they would be during play, and the coach makes play adjustments and motivates players. The team then re-enters the pitch and starts playing. Somewhere in this sequence of events a home-team advantage is created if it has scored just before the halftime. We view the motivational effect as the most likely source.

Our findings and the sentiment of football players and coaches can be combined to form theoretical implications. There is indeed an emotional impact of performance outcomes, and this impact is processed and stored differently when it is considered during a break and when there is no subsequent break, but rather continued effort without close interaction of team and coach. The gain in motivation for a team that has just received positive feedback through scoring a goal can indeed lead to improved performance and a win through a cognitive confirmation bias or through conservation of emotions [5–8]. This finding is important theoretically because there is yet little work on how such motivational effects linger under some circumstances and dissipate under other circumstances. More research should be conducted to examine this relationship.

There are also implications to related situations in soccer. The players’ and coaches’ emphasis on a mental boost are related to another old soccer wisdom. It is commonly believed that a team is particularly vulnerable to being scored against in the first few minutes after having scored a goal. The underlying reasoning is again the mental boost of the scoring team, which can lead to less cautious play. We are not aware of tests of this effect, but if it were found to be true, it would add to a body of evidence that successes yield confidence and some degree of inadvertent risk taking, for better or for worse.

The finding has applied implications for sports, and more generally for organizations with easily measurable performance outcomes. If the performance in the preceding active period after a success is a trade-off between the mental boost (being offensive, aggressive) and being over-confident (systematically underestimating risk), but a cool-off period (a break) will preserve more of the mental boost than the over-confidence, then it suggests it is tactically wise—in businesses and sports where striking a balance between being aggressive and defensive in decision making is essential—to reward success with breaks. Would rewarding decision making employees’ success with an instant holiday instead of a financial bonus be more beneficial for the company? This counter-intuitive approach (in basketball and handball normally players who have been making poor shot-decisions are given a break, not the “hot hands”) may somewhat cool off the hands of hot-handed decision makers, but—paying more dividend if the above is true—cool down their eagerness to go for too risky shots.

Within the field of management, one of the best-known research streams on this effect documents top management hubris, or belief in own infallibility [21, 22]. Within the field of finance, it is well-documented that prior success in investing leads to over-confidence and increased risk-taking among regular and professional investors [23, 24]. Both of these findings can be connected to research such as ours because they may be instances of decision-makers experiencing the same kind of mental boost as soccer players, either for a short time or for a longer duration. In both management and finance, the economic consequences of the resulting behaviors are significant, as they have been related to significant risk taking and losses in actions such as mergers and acquisitions (of firms) and transactions in financial markets.

One caution is that we cannot determine whether our findings originate in the scoring team or the conceding team. However, success as a temporary mental boost is well-documented in general, so in that respect soccer players are not special. Loss of motivation after disappointments is also a general effect. An interesting feature of the halftime effect that could be applicable broadly is that a break after a confidence-boosting event seems to be beneficial. If this also holds true for other types of work, a counter-intuitive implication is that a recently successful decision maker will do better when forced to have a cooling off period before making more decisions involving risk. Such practices may also be beneficial beyond sports, such as in the management and finance.

More broadly, our findings suggest a need for new research with a different focus than past work. Researchers have learnt much about the effects of failure on the human mind [25, 26], so it is now time for more research on the effects of success.

## Supporting information

S1 Appendix (DOCX)

S1 Table (DOCX)

S2 Table (DOCX)
