# Supplementary material for: Are goals scored just before halftime worth more? An old soccer wisdom statistically tested
Source: PLoS One. 2020 Oct 20;15(10):e0240438. doi: 10.1371/journal.pone.0240438 (PMC7575079; doi:10.1371/journal.pone.0240438)
Supplement: S1 Appendix — (DOCX) [file pone.0240438.s001.docx]

**S1 Appendix: Top Leagues Used in Analysis (WF-WFC)**

| **Country** | **League** | **Matches** | **Home Goals** | **Away Goals** |
| --- | --- | --- | --- | --- |
| Argentina | Primera División | 3665 | 1.37 | 1.03 |
| Belgium | Eerste klasse A | 2150 | 1.72 | 1.17 |
| Brazil | Série A | 2932 | 1.57 | 1.06 |
| Colombia | Primera A | 4520 | 1.65 | 1.22 |
| Denmark | Superliga | 1550 | 1.47 | 1.01 |
| England | Premier League | 2601 | 1.53 | 1.23 |
| Spain | Primera División | 5416 | 1.53 | 1.12 |
| Germany | Bundesliga | 5691 | 1.6 | 1.13 |
| Finland | Veikkausliiga | 645 | 1.55 | 1.21 |
| France | Ligue 1 | 3142 | 1.38 | 0.95 |
| Greece | Superleague | 2102 | 1.39 | 0.93 |
| Ireland | Premier Division | 1181 | 1.44 | 1.16 |
| Israel | Ligat ha'Al | 1141 | 1.41 | 1.12 |
| Italy | Serie A | 4832 | 1.52 | 1.11 |
| Mexico | Primera División | 1851 | 1.59 | 1.2 |
| Netherlands | Eredivisie | 4367 | 1.75 | 1.26 |
| Norway | Eliteserien | 2302 | 1.76 | 1.28 |
| Peru | Primera División | 1831 | 1.61 | 0.96 |
| Poland | Ekstraklasa | 1454 | 1.4 | 1.01 |
| Portugal | Primeira Liga | 2961 | 1.47 | 1.06 |
| South Africa | Premier Division | 1419 | 1.27 | 1.03 |
| Russia | Premier Liga | 2478 | 1.4 | 1.06 |
| Scotland | Premiership | 3162 | 1.49 | 1.22 |
| Sweden | Allsvenskan | 2320 | 1.58 | 1.18 |
| Turkey | SüperLig | 3985 | 1.55 | 1.16 |
| Ukraine | Premyer Liga | 1396 | 1.41 | 1.11 |
| Uruguay | Primera División | 1332 | 1.46 | 1.32 |
