# Supplementary material for: Are goals scored just before halftime worth more? An old soccer wisdom statistically tested
Source: PLoS One. 2020 Oct 20;15(10):e0240438. doi: 10.1371/journal.pone.0240438 (PMC7575079; doi:10.1371/journal.pone.0240438)
Supplement: S1 Table — (DOCX) [file pone.0240438.s002.docx]

**S1 Table: Descriptive statistics for datasets**

|  | **Dataset** | | | | | | | | | | | | | |
| --- | --- | --- | --- | --- | --- | --- | --- | --- | --- | --- | --- | --- | --- | --- |
|  | **BA**  **(N = 1,179)** | | **WF-BA**  **(N = 1,563)** | | **WF-ODDS**  **(N = 167,912)** | | **WF-L**  **(N = 171,949)** | | **WF-LT**  **(N = 122,786)** | | **WFC**  **(N = 72,426)** | | **WF-MAX93**  **(N = 305,942)** | |
|  | **Mean** | **SD** | **Mean** | **SD** | **Mean** | **SD** | **Mean** | **SD** | **Mean** | **SD** | **Mean** | **SD** | **Mean** | **SD** |
| **Goal difference at full time** | -0.39 | 1.83 | -0.41 | 1.86 | -0.37 | 1.82 | -0.39 | 1.71 | -0.42 | 1.72 | -0.41 | 1.73 | -0.37 | 1.87 |
| **Victory by home team** | 0.46 | 0.50 | 0.47 | 0.50 | 0.46 | 0.50 | 0.46 | 0.50 | 0.46 | 0.50 | 0.47 | 0.50 | 0.46 | 0.50 |
| **Victory by away team** | 0.29 | 0.46 | 0.29 | 0.45 | 0.29 | 0.45 | 0.27 | 0.45 | 0.27 | 0.44 | 0.27 | 0.45 | 0.29 | 0.45 |
| **Final number of goals by home team** | 1.53 | 1.30 | 1.54 | 1.33 | 1.55 | 1.33 | 1.51 | 1.27 | 1.53 | 1.29 | 1.53 | 1.29 | 1.56 | 1.35 |
| **Final number of goals by away team** | 1.14 | 1.14 | 1.13 | 1.14 | 1.17 | 1.17 | 1.12 | 1.11 | 1.11 | 1.11 | 1.13 | 1.11 | 1.19 | 1.20 |
| **Goal home team between start of minute 45 and half time** | 0.04 | 0.20 | 0.04 | 0.19 | 0.04 | 0.19 | 0.04 | 0.19 | 0.04 | 0.19 | 0.04 | 0.19 | 0.04 | 0.19 |
| **Goal away team between start of minute 45 and half time** | 0.04 | 0.19 | 0.04 | 0.18 | 0.03 | 0.17 | 0.03 | 0.16 | 0.03 | 0.16 | 0.03 | 0.16 | 0.03 | 0.16 |
| **Goal home team between start of minute 44 and half time** | 0.05 | 0.22 | 0.05 | 0.22 | 0.06 | 0.23 | 0.05 | 0.23 | 0.05 | 0.23 | 0.06 | 0.23 | 0.05 | 0.23 |
| **Goal away team between start of minute 44 and half time** | 0.05 | 0.21 | 0.05 | 0.21 | 0.04 | 0.20 | 0.04 | 0.19 | 0.04 | 0.19 | 0.04 | 0.19 | 0.04 | 0.20 |
| **Goal home team between start of minute 43 and half time** | 0.07 | 0.25 | 0.06 | 0.24 | 0.07 | 0.26 | 0.07 | 0.25 | 0.07 | 0.26 | 0.07 | 0.26 | 0.07 | 0.26 |
| **Goal away team between start of minute 43 and half time** | 0.06 | 0.23 | 0.06 | 0.23 | 0.05 | 0.22 | 0.05 | 0.22 | 0.05 | 0.22 | 0.05 | 0.22 | 0.05 | 0.22 |
| **Goal home team between start of minute 42 and half time** | 0.08 | 0.27 | 0.08 | 0.27 | 0.09 | 0.28 | 0.08 | 0.28 | 0.08 | 0.28 | 0.09 | 0.28 | 0.08 | 0.28 |
| **Goal away team between start of minute 42 and half time** | 0.07 | 0.25 | 0.07 | 0.25 | 0.06 | 0.25 | 0.06 | 0.24 | 0.06 | 0.24 | 0.06 | 0.24 | 0.06 | 0.25 |
| **Goal home team between start of minute 41 and half time** | 0.09 | 0.29 | 0.10 | 0.29 | 0.10 | 0.30 | 0.10 | 0.30 | 0.10 | 0.30 | 0.10 | 0.30 | 0.10 | 0.30 |
| **Goal away team between start of minute 41 and half time** | 0.08 | 0.27 | 0.08 | 0.27 | 0.08 | 0.26 | 0.07 | 0.26 | 0.07 | 0.26 | 0.07 | 0.26 | 0.08 | 0.26 |
| **Goal difference at half time** | -0.19 | 1.14 | -0.18 | 1.15 | -0.17 | 1.12 | -0.18 | 1.08 | -0.19 | 1.08 | -0.19 | 1.09 | -0.17 | 1.14 |
| **Number of goals home team during first half** | 0.68 | 0.85 | 0.67 | 0.84 | 0.68 | 0.84 | 0.67 | 0.82 | 0.68 | 0.82 | 0.68 | 0.82 | 0.69 | 0.85 |
| **Last goal in first half by home team** | 0.39 | 0.49 | 0.39 | 0.49 | 0.40 | 0.49 | 0.40 | 0.49 | 0.40 | 0.49 | 0.40 | 0.49 | 0.40 | 0.49 |
| **Last goal in first half by away team** | 0.28 | 0.45 | 0.28 | 0.45 | 0.30 | 0.46 | 0.29 | 0.45 | 0.28 | 0.45 | 0.29 | 0.45 | 0.30 | 0.46 |
| **Goal difference (2nd halftime only)** | -0.20 | 1.30 | -0.23 | 1.32 | -0.20 | 1.32 | -0.21 | 1.27 | -0.22 | 1.27 | -0.22 | 1.28 | -0.20 | 1.35 |
| **Home team goal arrival rate (estimated from betting odds)** |  |  |  |  | 1.35 | 0.43 |  |  |  |  | 1.32 | 0.41 |  |  |
| **Away team goal arrival rate (estimated from betting odds)** |  |  |  |  | 1.02 | 0.36 |  |  |  |  | 0.96 | 0.32 |  |  |
| **Game in UEFA Europa League** | 0.44 | 0.50 | 0.52 | 0.50 | 0.01 | 0.11 | 0.00 | 0.00 | 0.00 | 0.00 | 0.00 | 0.00 | 0.01 | 0.12 |
| **Game in group phase** | 0.70 | 0.46 |  |  |  |  |  |  |  |  |  |  |  |  |
