# Supplementary material for: Are goals scored just before halftime worth more? An old soccer wisdom statistically tested
Source: PLoS One. 2020 Oct 20;15(10):e0240438. doi: 10.1371/journal.pone.0240438 (PMC7575079; doi:10.1371/journal.pone.0240438)
Supplement: S2 Table — (DOCX) [file pone.0240438.s003.docx]

**S2 Table: Score difference analysis, multiple data sets**

|  | End of game score difference (away goals - home goals) | | | | | | | | | | | | |
| --- | --- | --- | --- | --- | --- | --- | --- | --- | --- | --- | --- | --- | --- |
|  | BA | | WF-BA | | WF-ODDS | | | WF-L | WF-LT | WFC | WF-MAX93 | | |
|  | (1) | (2) | (3) | (4) | (5) | (6) | (7) | (8) | (9) | (10) | (11) | (12) | (13) |
| Late home goal | 0.230 | 0.261 | -0.040 | -0.009 | -0.028^*^ | -0.042^***^ | -0.037^***^ | -0.059^***^ | -0.065^***^ | -0.054^**^ | -0.057^***^ | -0.049^***^ | -0.036^***^ |
|  | (0.176) | (0.180) | (0.158) | (0.160) | (0.015) | (0.015) | (0.014) | (0.014) | (0.017) | (0.022) | (0.011) | (0.011) | (0.011) |
| Late away goal | 0.170 | 0.123 | 0.209 | 0.176 | -0.009 | 0.009 | 0.018 | 0.025 | 0.033^*^ | 0.011 | 0.031^**^ | 0.029^**^ | 0.009 |
|  | (0.185) | (0.193) | (0.165) | (0.170) | (0.017) | (0.017) | (0.016) | (0.017) | (0.020) | (0.026) | (0.013) | (0.012) | (0.013) |
| Score difference | 1.118^***^ | 1.074^***^ | 1.150^***^ | 1.143^***^ | 1.125^***^ | 1.142^***^ | 1.006^***^ | 1.064^***^ | 1.066^***^ | 1.062^***^ | 1.194^***^ | 1.047^***^ |  |
|  | (0.053) | (0.081) | (0.047) | (0.070) | (0.005) | (0.007) | (0.006) | (0.007) | (0.008) | (0.010) | (0.005) | (0.005) |  |
| Number of home | -0.050 | -0.055 | 0.009 | 0.054 | 0.013^**^ | 0.007 | 0.003 | -0.007 | -0.019^*^ | -0.022^*^ | 0.028^***^ | 0.008 |  |
| goals | (0.071) | (0.106) | (0.064) | (0.095) | (0.006) | (0.009) | (0.008) | (0.009) | (0.010) | (0.013) | (0.006) | (0.006) |  |
| Game in UEFA |  | -0.069 |  |  |  |  |  |  |  |  |  |  |  |
| Europe League |  | (0.076) |  |  |  |  |  |  |  |  |  |  |  |
| Game in group |  | 0.044 |  |  |  |  |  |  |  |  |  |  |  |
| phase |  | (0.083) |  |  |  |  |  |  |  |  |  |  |  |
| λ_h_ |  |  |  |  |  |  | -0.655^***^ |  |  |  |  |  |  |
|  |  |  |  |  |  |  | (0.009) |  |  |  |  |  |  |
| λ_a_ |  |  |  |  |  |  | 0.659^***^ |  |  |  |  |  |  |
|  |  |  |  |  |  |  | (0.010) |  |  |  |  |  |  |
| Home scored last |  | -0.090 |  | -0.135 |  | 0.050^***^ | 0.043^***^ | 0.033^***^ | 0.045^***^ | 0.046^***^ | 0.067^***^ | 0.051^***^ |  |
|  |  | (0.133) |  | (0.120) |  | (0.011) | (0.011) | (0.011) | (0.013) | (0.017) | (0.009) | (0.008) |  |
| Away scored last |  | 0.067 |  | -0.018 |  | -0.023^*^ | -0.020^*^ | 0.0003 | 0.009 | 0.020 | -0.058^***^ | -0.026^***^ |  |
|  |  | (0.154) |  | (0.133) |  | (0.012) | (0.012) | (0.012) | (0.014) | (0.019) | (0.009) | (0.009) |  |
| Constant | -0.162^***^ | -0.150 | -0.214^***^ | -0.188^***^ | -0.189^***^ | -0.196^***^ | 0.001 | -0.204^***^ | -0.215^***^ | -0.215^***^ | -0.195^***^ |  |  |
|  | (0.055) | (0.097) | (0.048) | (0.058) | (0.005) | (0.006) | (0.020) | (0.005) | (0.006) | (0.009) | (0.004) |  |  |
| Team FE |  |  |  |  |  |  |  |  |  |  |  | Yes | Yes |
| Competition FE |  |  |  |  |  |  |  |  |  |  |  | Yes | Yes |
| Goal sequence FE |  |  |  |  |  |  |  |  |  |  |  |  | Yes |
| Observations | 1,179 | 1,179 | 1,563 | 1,563 | 167,912 | 167,912 | 167,912 | 171,949 | 122,786 | 72,426 | 305,942 | 305,422 | 305,422 |
| R^2^ | 0.505 | 0.506 | 0.506 | 0.506 | 0.475 | 0.475 | 0.533 | 0.451 | 0.454 | 0.453 | 0.489 | 0.554 | 0.555 |
| Adjusted R^2^ | 0.503 | 0.503 | 0.504 | 0.504 | 0.475 | 0.475 | 0.533 | 0.451 | 0.454 | 0.453 | 0.489 | 0.541 | 0.542 |
| Note: | *p<0.1; *****p<0.05;*** ***p<0.01 | | | | | | | | | | | | |
